# Amphibian Diversity and Threatened Species in a Severely Transformed Neotropical Region in Mexico

**DOI:** 10.1371/journal.pone.0121652

**Published:** 2015-03-23

**Authors:** Yocoyani Meza-Parral, Eduardo Pineda

**Affiliations:** Red de Biología y Conservación de Vertebrados, Instituto de Ecología, A.C., Xalapa, Veracruz, México; Trier University, GERMANY

## Abstract

Many regions around the world concentrate a large number of highly endangered species that have very restricted distributions. The mountainous region of central Veracruz, Mexico, is considered a priority area for amphibian conservation because of its high level of endemism and the number of threatened species. The original tropical montane cloud forest in the region has been dramatically reduced and fragmented and is now mainly confined to ravines and hillsides. We evaluated the current situation of amphibian diversity in the cloud forest fragments of this region by analyzing species richness and abundance, comparing assemblage structure and species composition, examining the distribution and abundance of threatened species, and identifying the local and landscape variables associated with the observed amphibian diversity. From June to October 2012 we sampled ten forest fragments, investing 944 person-hours of sampling effort. A total of 895 amphibians belonging to 16 species were recorded. Notable differences in species richness, abundance, and assemblage structure between forest fragments were observed. Species composition between pairs of fragments differed by an average of 53%, with the majority (58%) resulting from species replacement and the rest (42%) explained by differences in species richness. Half of the species detected are under threat of extinction according to the International Union for Conservation of Nature, and although their distribution and abundance varied markedly, there were also ubiquitous and abundant species, along with rare species of restricted distribution. The evident heterogeneity of the ten study sites indicates that to conserve amphibians in a mountainous region such as this one it is necessary to protect groups of fragments which represent the variability of the system. Both individually and together cloud forest fragments are very important to conservation because each remnant is inhabited by several threatened species, some of them at imminent risk of extinction.

## Introduction

Several sites around the world are home to a large number of highly endangered species, the distribution of which is very restricted. These sites, mainly located in tropical forest, on islands or in mountainous areas, are of particular concern because most of them are located in regions that are severely impacted by human activities and only one-third are protected by law [1]. Amphibians have been the focus of attention around the world in the context of conservation because recent evaluations estimate that one in three of the amphibian species on the planet is endangered [2,3]. Amphibians represent 51% of the threatened species that inhabit the sites that host species in imminent danger of extinction (including mammals, birds, reptiles and conifers) [1].

The mountainous region of central Veracruz, in eastern Mexico, is considered a priority area for amphibian conservation, not only for this country [4,5] but also globally, since half of the species that have been recorded there are threatened according to the IUCN [3] and this exceeds the world average. Six of the species that inhabit tropical montane cloud forest (TMCF) are in imminent danger of extinction according to the Alliance for Zero Extinction [6] and several species have been targeted by the Evolutionarily Distinct and Globally Endangered (EDGE) program that highlights and seeks to conserve one-of-a-kind species that are on the verge of extinction (edgeofexistence.org). The region is also exceptionally diverse in salamanders because it contains representatives of five genera: *Bolitoglossa*, *Chiropterotriton*, *Parvimolge*, *Pseudoeurycea* and *Thorius* [7].

Tropical montane cloud forest originally covered most of central Veracruz and has undergone a drastic reduction in the last century. It is estimated that only 7% of primary TMCF (i.e., that which has a low degree of anthropogenic disturbance) remains in the region, and there is an additional 10% present as secondary forest (with disturbance but showing some degree of recovery). Thus, more than 80% of the area is currently dominated by transformed environments such as coffee plantations, cattle pastures, sugarcane crops and human settlements, among other types of land use [8]. The TMCF remnants, both conserved and secondary, are mainly confined to ravines, canyons and hillsides [9].

Under this scenario, TMCF fragments may offer environmental conditions for the maintenance of a variety of amphibian species of the region, even those that are threatened. However, given the heterogeneity of this Neotropical mountain system, along with the environmental changes induced by landscape transformation, each fragment would likely provide different environmental conditions for amphibians, which we would expect to be reflected in differences in species richness, abundance and composition between forest fragments. Additionally, owing to their restricted distribution and high degree of sensitivity to habitat modification, threatened species would be expected to inhabit few forest fragments and their abundance would be expected to be low.

In this study we (1) examine amphibian species richness and abundance in individual TMCF fragments and for the fragments as a whole, (2) compare assemblage structure between fragments, (3) evaluate differences in species composition between fragments, (4) identify the distribution and abundance of the threatened species, and (5) examine which local and landscape variables are related to observed species richness, abundance and differences in composition. Thus, this study aims to contribute to a better understanding of the current situation of amphibians in a severely transformed region of high biological relevance, and to help determine the importance of TMCF fragments to the conservation of biodiversity at the regional level.

## Methods

### Ethics statement

To conduct this work we first obtained permits from the Mexican wildlife agency Dirección General de Vida Silvestre of the Secretaría de Medio Ambiente y Recursos Naturales to work with wild animals (collecting permit number: SGPA/DGVS/03665/ 06). Manipulation of animals in the field was minimal in all cases because we used a visual encounter survey method [10]. After identifying each amphibian collected, individuals were returned to the same site where they were found. Only the first individuals captured for each species were killed using lidocaine and preserved in 70% alcohol as voucher specimens, following the methods suggested for amphibians [11]. The collecting permit allowed for the capture of threatened species and the preservation in alcohol of the first specimens because the purpose of the study was exclusively scientific. For field sampling in Mexico, approval by an Institutional Animal Care and Use Committee (IACUC) or equivalent animal ethics committee is not required.

### Area and study sites

Fieldwork was carried out in the surroundings of Xalapa and Huatusco, in the state of Veracruz, Mexico (between 19°32'-19°04' N and 96°55'-97°05' W). The study area covers 1000 to 1900 m a.s.l. Mean annual temperature is 18°C (ranging on average from 14° to 22°C throughout the year), total annual precipitation ranges from 1600 to 2000 mm and there are three seasons: warm-dry (March-May), warm-humid (June-October) and cold-wet (November-February) [12]. It is estimated that the total area currently covered by TMCF remnants, both preserved and disturbed, is about 130,000 ha [9].

**Fig 1:**
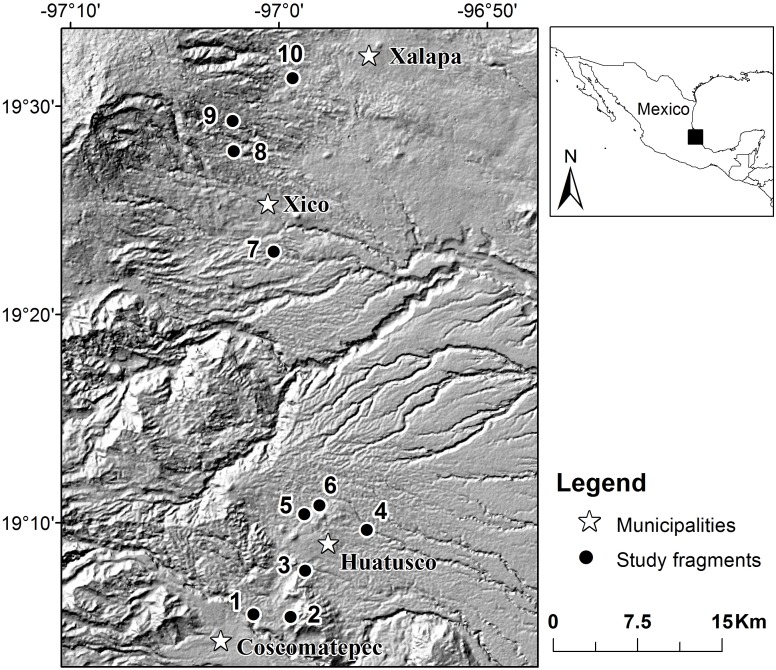
Location of ten study sites in the mountainous region of central Veracruz in Mexico.

Ten forest fragments were selected as study sites based on an analysis of digital aerial images of Google Earth [13] and we hiked through each forest fragment to confirm site suitability. The criteria for selection were that: 1) each site should be located in a TMCF fragment with little disturbance, 2) each fragment must have at least one stream to ensure that the absence of water was not a limiting factor for the amphibian fauna that require water to complete their life cycle, and 3) the distance between fragments should be greater than 1.5 km to ensure the independence of samples in the measurement of local diversity. The ten study fragments were located along a 48-km-long straight line (distance between the farthest forest fragments) and the minimum distance between fragments was 1.9 km (Fig. 1).

### Data collection

#### Amphibian sampling

Amphibians were collected from all possible microhabitats during direct searches (visual and auditory) of the ten study sites, and using a time-constrained technique [10]. From June to October 2012, each site was visited four times and during each visit four people searched for about six hours (four hours at night and two hours during the day). Specimens collected (postmetamorphic phase only) were identified and then released at the point of capture. Amphibians that were difficult to identify in the field were taken to the laboratory to verify their identity using the relevant literature. Total sampling effort was 944 person-hours.

#### Habitat assessment

At each study site we set up ten 4 × 25 m plots (total area: 0.1 ha) in which we counted the trees with a diameter at breast height (DBH) >5 cm, measured their diameter, visually estimated their height and measured the depth of the litter layer at 15 points (5 at one end of the plot, 5 in the middle and 5 at the other end; 150 per site). Additionally, in each plot we measured canopy cover by taking three digital photographs of the canopy (one from each side of the plot and one from the center) from 1.3 m above the ground. The digital images were processed following the method proposed by Korhonen et al. [14] using ImageJ software version 1.43 [15] to calculate percentage canopy cover. In each plot we also recorded whether there were any signs of human disturbance such as solid waste, logging or burning.

The area and perimeter of each fragment was measured using images from Google Earth version 6.1 [13] and analyzed with ArcGIS software v.10.0 [16]. The shape of the fragment (S) was calculated as $S=P/2\sqrt{\pi A}$, where *P* is the perimeter of the forest fragment and *A* is the area [17]. The minimum value of *S* is 1 when the shape of the site is a perfect circle and this value increases as the shape of the site becomes more irregular. The characteristics of the study sites are summarized in Table 1.

**Table 1:** Characteristics of ten forest fragments in the mountainous region of central Veracruz, Mexico.

Site	N Latitude	W Longitude	Elevation (m a.s.l.)	Size (km²)	Shape	Canopy cover (%)	Tree density (in 1000 m²)	Basal area (in 1000 m²)	Tree height ± SD (m)	Leaf litter depth ± SD (cm)
1	19.10	-97.03	1305	0.84	3.17	85.1	143	10.71	14±9	5±3
2	19.09	-97.00	1407	1.24	3.41	88.3	173	9.04	14±8	6±3
3	19.12	-96.98	1269	0.71	4.35	83.4	76	13.12	12±9	5±3
4	19.16	-96.93	1126	0.09	1.31	87.8	128	6.46	11±7	4±3
5	19.17	-96.97	1299	0.68	3.05	87.3	123	12.38	14±10	6±3
6	19.18	-96.96	1269	0.38	3.64	85.0	179	10.73	12±8	5±4
7	19.38	-97.00	1189	0.24	2.06	85.9	144	5.66	13±8	5±3
8	19.45	-97.03	1927	1.65	3.46	88.0	182	9.71	14±9	7±4
9	19.48	-97.02	1915	3.53	3.95	94.4	140	8.67	12±8	7±3
10	19.52	-96.98	1448	0.27	1.68	80.2	168	17.46	15±11	5±3

#### Data analysis

Species richness was defined as the number of species recorded during the entire study period and abundance was the number of amphibians detected. To assess the level of inventory completeness for each fragment and in all sites together we used the Chao 1 and Mao Tau estimators plus their 84% confidence intervals [18]. Species estimators were calculated using EstimateS v 9.1.0 software [19]. To compare species richness among fragments we used the Mao Tau species estimator value and its 84% confidence interval. Because EstimateS generates 95% confidence intervals for each species estimator value, we multiplied its associated SD by the quantile (z-score of the normal curve) corresponding to two-sided intervals of 84% probabilities (1.372). As suggested by MacGregor-Fors & Payton [18], values were interpreted as statistically different when the 84% confidence intervals did not overlap, and not different when they did overlap, at α = 0.05. Comparisons were made of the same sampling effort (in this case the number of amphibians), extrapolating estimates and using the site with the highest abundance recorded as a reference value (see Colwell et al. [20]).To compare the distribution of amphibian abundance among sites we used a χ^2^ test [21]. Assemblage structure was compared among sites using dominance-diversity graphs [22].

To analyze differences in species composition among fragments we applied the method proposed by Carvalho et al. [23]. The compositional differences between two sites can be partitioned into two components: species replacement and species richness differences, in an additive way: Difference in composition = replacement + difference in species richness.

This approach allows us to identify the different causes of patterns in compositional dissimilarity along ecological gradients by estimating the relative contributions of replacement and differences in richness.

To this end, an overall measure of compositional dissimilarity between two sites is obtained using the Jaccard dissimilarity index (denoted as β_cc_ as Carvalho et al. [23] suggests):

$$\beta_{\text{cc}}=\frac{b+c}{a+b+c}$$

Where *a* is the number of species in both sites, *b* is the number of species occurring in the first site but not in the second, and *c* is the number of species in the second site but not in the first.

Replacement between two sites is the substitution of *n* species in a given site by *n* species in another site. The measure (denoted as β_-3_) can be obtained calculating:

$$\beta_{-3}=2\times \frac{\text{min}(b,c)}{a+b+c}$$

Where min(*b*,*c*) is the minimum number of exclusive species in one of the two sites.

To obtain the difference in species richness between two sites, we used the measure:

$$\beta_{\text{rich}}=\frac{|b-c|}{a+b+c}$$

The distribution and abundance of endangered species in the study sites were obtained using species classified as critically endangered (CR), endangered (EN) and vulnerable (VU) according to the Red List of Threatened Species managed by the International Union for Conservation of Nature [24].

A multiple regression model (backward stepwise) was used to identify the habitat traits (Table 1) related to amphibian species richness and abundance. Prior to running the analyses, the dependent variables were square root transformed. Data were analyzed using the software Statistica version 7.0 [25]. To detect whether dissimilarity in composition, replacement or differences in species richness were related to differences in habitat attributes (Euclidean distances), or to the geographic distance between forest fragments, we used the Mantel test [26]. Data were analyzed using PASSaGE software v. 2 [27] with 999 permutations in each analysis.

## Results

### Species richness and abundance

**Fig 2:**
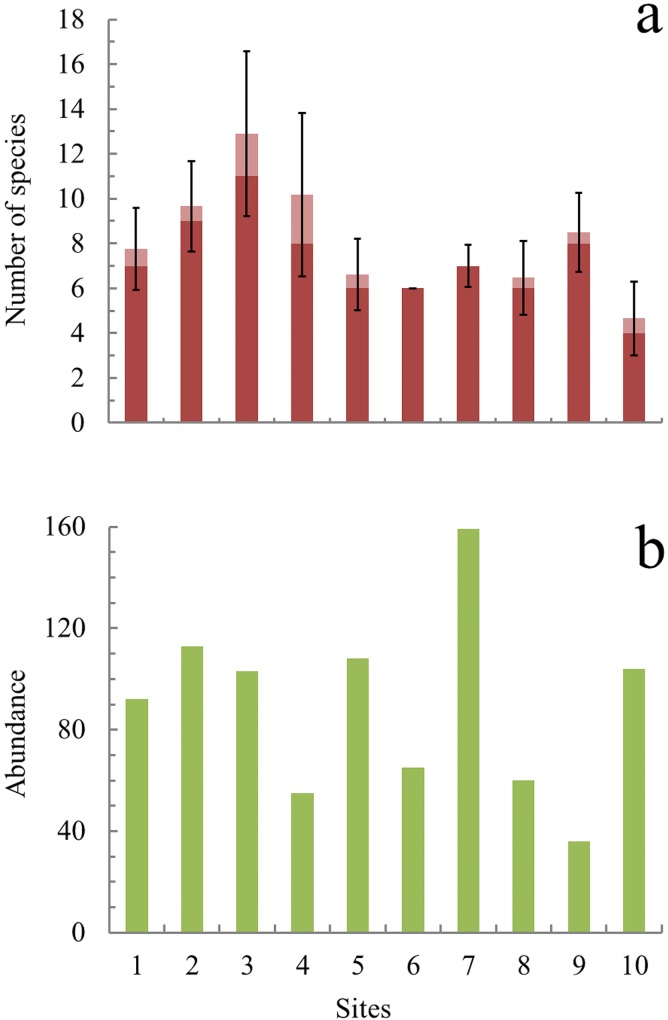
Amphibian species richness and abundance in ten study sites in central Veracruz, Mexico. a) Observed (dark red) and estimated species richness (light red). The error bars indicate the upper and the lower Confidence Intervals at 84%. Comparisons of estimated species richness and Confidence Intervals were made with the same sampling effort (number of individuals), extrapolating estimates and using site 7 as a reference value because it had the highest abundance recorded. b) Cumulative abundance for each study site.

Across all surveys a total of 16 amphibian species (12 anuran species and four salamander species) belonging to seven families were recorded (Table 2). Among TMCF fragments, species richness differed markedly, ranging from four species (Site 10) to 11 (Site 3) (Fig. 2A). Total amphibian abundance was 895 amphibians, with notable differences between forest fragments (*χ* ^*2*^ = 130, *d*.*f*. = 9, *p*<0.0001): from 36 at site 9 to 159 at site 7 (Fig. 2B).

**Table 2:** Amphibian species recorded in ten sites (TMCF fragments), their abundance and IUCN category* in central Veracruz, Mexico.

		Site										Total	
Code	Species	1	2	3	4	5	6	7	8	9	10		IUCN
	CRAUGASTORIDAE												
A	*Craugastor decoratus*		2	1						3		6	VU
B	*Craugastor rhodopis*	17	30	12	1	64	19	65	24	4	43	279	VU
C	*Craugastor mexicanus*									2		2	LC
D	*Craugastor pygmaeus*			4	3							7	VU
	ELEUTHERODACTYLIDAE												
E	*Eleutherodactylus cystignathoides*			3	12	1	5	2				23	LC
	CENTROLENIDAE												
F	*Hyalinobatrachium fleischmanni*		5	16	15	4	9	10				59	LC
	BUFONIDAE												
G	*Incilius cristatus*	1	3									4	CR
H	*Incilius valliceps*			1	1							2	LC
	HYLIDAE												
I	*Charadrahyla taeniopus*	1	1	23		1	7	10	2		59	104	VU
J	*Ecnomiohyla miotympanum*	61	61	39	20	26	22	68	1	3		301	NT
K	*Plectrohyla arborescandens*								16	14		30	EN
	RANIDAE												
L	*Lithobates berlandieri*	8	1	1				1				11	LC
	PLETHODONTIDAE												
M	*Chiropterotriton sp*.									1		1	NE
N	*Parvimolge townsendi*	2	7	2	1	12	3	3		4	1	35	CR
O	*Pseudoeurycea cafetalera*	2	3	1	2				14	5	1	28	NE
P	*Pseudoeurycea lynchi*								3			3	CR
	Abundance	92	113	103	55	108	65	159	60	36	104	895	
	Number of species	7	9	11	8	6	6	7	6	8	4	16	

* CR = Critically Endangered, EN = Endangered, VU = Vulnerable, LC = Least Concern, NT = Near Threatened, NE = Not Evaluated.

Inventory completeness for the set of study sites, according to species richness estimators is between 93% and 100%. There was just one singleton (species represented by just one individual) and two doubletons (species represented by two individuals) (see S1 Fig.). At the fragment level inventory completeness ranged from 79% to 100%, with sites 6, 7, 8 and 9 having the highest degree of completeness and site 3 the lowest (see S1 Table).

### Assemblage structure

**Fig 3:**
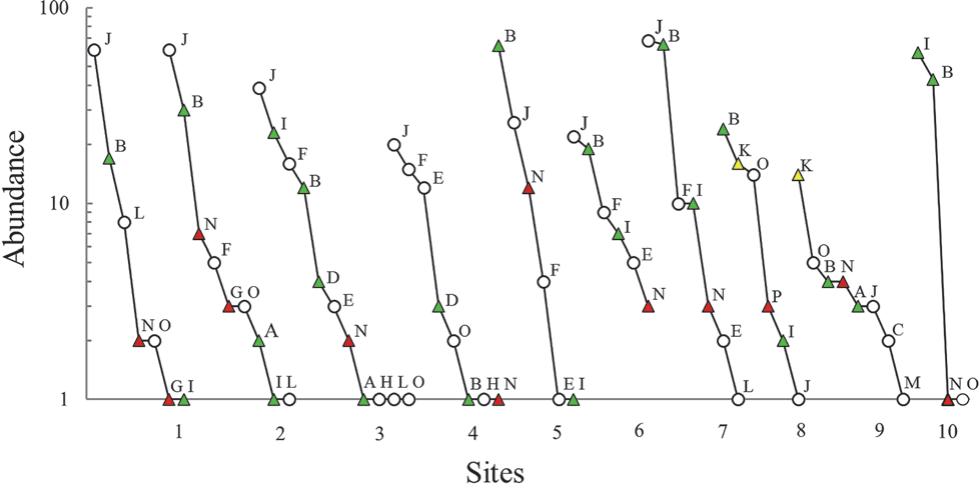
Dominance-diversity graphs of amphibian assemblages in ten TMCF fragments in central Veracruz, Mexico. Letters denote species (see Table 2), numbers on the X axis are site numbers, green triangles indicate Vulnerable species, yellow triangles are Endangered species, red triangles are Critically endangered species, and circles are species that are not threatened or were not evaluated.

The slope of the dominance-diversity graphs varied between study sites (Fig. 3). For example, the slopes of sites 3, 6 and 9 were lower than those of sites 1, 2, 5 and 7, and much lower than that of site 10. Thus, in the first sites (3, 6 and 9) evenness was greater than in the remaining sites. One species was dominant in six of the ten study sites but was not recorded in one site (10) (Fig. 3). In almost all assemblages, except that of site 6, there were rare species (i.e., those with just one individual); the number varying between four and one species. The salamander *Chiropterotriton* sp. was the only species with only one individual in the study. The hierarchical position of the remaining species varied between forest fragments.

### Differences in species composition

**Fig 4:**
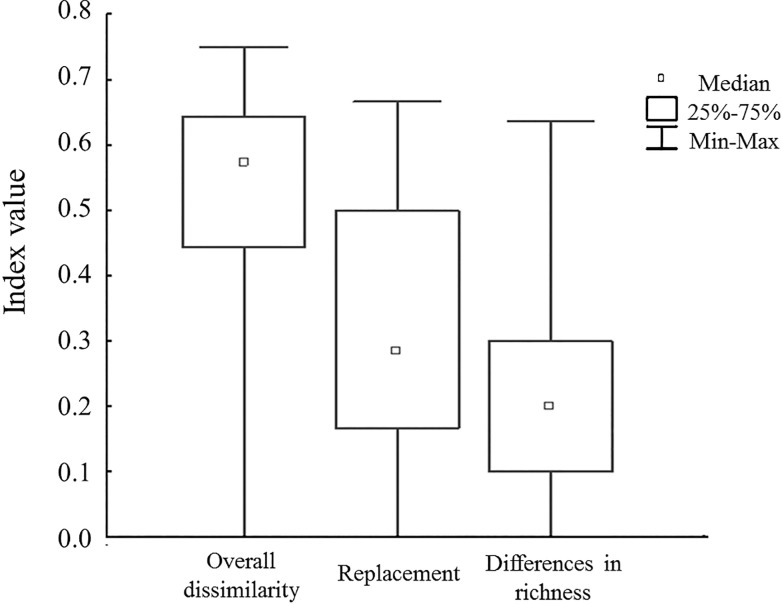
Compositional dissimilarity values between pairs of sites. Overall dissimilarity (β_cc_), replacement (β-_3_), and differences in species richness (β_rich_) are shown separately.

The overall compositional dissimilarity (β_cc_) between sites was 0.53 on average, ranging from 0.75 to zero. Replacement (β_-3_) between sites was 0.30 on average, equivalent to 58% of the total dissimilarity, and ranged from 0.67 (between four pairs of sites) to zero (between 11 pairs of sites). The difference in species richness (β_rich_) was 0.22 on average, equal to 42% of the overall compositional dissimilarity, and ranged from 0.64 (between two sites) to zero (between five pairs of sites) (see Fig. 4 and S2 Table for details).

### Distribution and abundance of threatened species

Fifty percent of the 16 amphibian species recorded in this study are threatened. Four species (*Craugastor decoratus*, *Craugastor rhodopis*, *Craugastor pygmaeus* and *Charadrahyla taeniopus*) are listed as vulnerable (VU), one species (*Plectrohyla arborescandens*) is endangered (EN) and three species *(Incilius cristatus*, *Parvimolge townsendi* and *Pseudoeurycea lynchi)* are critically endangered (CR) (Table 2). All of the study fragments harbored threatened species (between three and five species); indeed, in practically all of the sites 50% or more of the species that formed the assemblages were threatened. The distribution and abundance of threatened species across forest fragments varied markedly (Fig. 3). The litter frog *Craugasor rhodopis* was recorded in all fragments (and commonly in high abundances), the pygmy salamander *Parvimolge townsendi* (also a priority species for protection according to the Alliance for Zero Extinction) was detected in nine fragments, though its abundance was low, and the tree frog *Charadrahyla taeniopus* was in eight sites and had a highly variable abundance. In contrast, there were threatened species that occurred in just one site, such as *P*. *lynchi*, three species only found in two sites (*I*. *cristatus*, *P*. *arborescandens* and *C*. *pygmaeus*) and one species (*C*. *decoratus*) detected in three forest fragments (Fig. 3).

### Habitat traits and amphibian diversity

The multiple regression analysis (coefficient value given in parenthesis) revealed that species richness was directly related to fragment area (6.38), and inversely related to tree height (-0.75) and to the interaction between elevation and area (-6.2) (*F*_3,6_ = 7.71, *p*<0.017, *R* ^2^ = 0.794). Amphibian abundance was directly related to tree height (0.601) and inversely related to elevation (-0.720) (*F*_2, 7_ = 6.24, *p*<0.027, *R* ^2^ = 0.641). When abundance was analyzed at the family level (using only the most abundant families), the abundance of Hylidae was found to be negatively related to elevation (-0.72) (*F*_1,8_ = 8.54, *p*<0.019, *R* ^2^ = 0.516), while the abundance of Plethodontidae salamanders was related to leaf litter depth (0.839) (*F*_1,8_ = 19.06, *p*<0.0024, *R* ^2^ = 0.704). The abundance of litter frogs in Craugastoridae was inversely related to elevation (-1.5), canopy cover (-0.54), fragment shape (-0.76) and tree density (-0.64) (*F*_5,4_ = 8.29, *p*<0.030, *R* ^2^ = 0.912).

The Mantel test showed that species composition between two sites (β_cc_) tends to become increasingly different as the difference between sites in the following increases: elevation (Z_Mantel_ = 8444, p<0.001, r = 0.65), fragment area (Z_Mantel_ = 29, p = 0.015, r = 0.53), geographic distance (Z_Mantel_ = 592, p = 0.013, r = 0.37), and 4) leaf litter depth (Z_Mantel_ = 30, p = 0.025, r = 0.43). Species replacement (β_-3_) between pairs of sites tends to increase as the following increase: difference in elevation between sites (Z_Mantel_ = 5985, p<0.001, r = 0.72), difference in fragment area (Z_Mantel_ = 20.06, p = 0.018, r = 0.58), and difference leaf litter depth (Z_Mantel_ = 20, p = 0.016, r = 0.51). Finally, the difference in species richness (β_rich_) between any two sites tends to differ increasingly as the difference in the basal area of trees increases (Z_Mantel_ = 49, p = 0.039, r = 0.49) (see S3 Table).

## Discussion

Our results show that in a region as severely transformed as our study area, forest fragments offer suitable conditions for maintaining a portion of amphibian species of the region, including a significant number of threatened and critically endangered species. The remarkable variation in species richness, abundance, assemblage structure, and species composition between TMCF fragments highlights the heterogeneity of the mountainous region of central Veracruz, and suggests that to conserve amphibian species in areas like this, it is necessary to protect groups of forest fragments that are representative of the variability of the system. The conservation value of forest fragments is highlighted at both the individual and the collective level, because each remnant is inhabited by several threatened species, some of them at imminent risk of extinction.

The amphibian species recorded in this study within ten cloud forest fragments represent 37% of the 43 amphibian species historically recorded in the region (according to the following databases: the National Commission for the Knowledge and Use of Biodiversity database SNIB-CONABIO, GBIF [http://www.gbif.org] and HerpNet [http://www.herpnet.org]). Our surveys were carried out in forest fragments that together cover less than 1% of the TMCF that remains in the region. The species not recorded during this study may inhabit other forest fragments in the region, matrix habitats [28–30], may be present in the study fragments but in very low numbers, in non-sampled microhabitats such as bromeliads in the canopy, or they may have been buried during the sampling period because the fossorial habit of some species.

The variation in species richness, abundance, assemblage structure, and species composition among fragments suggests that each forest remnant offers particular conditions created by the inherent heterogeneity of the mountainous region and by differences in forest fragment traits. These conditions are differentially exploited by species or groups of species, even by those that are threatened.

Fragment area (size) has long been recognized as a fundamental attribute related to the number of species [31], but in our study, fragment size is also linked to elevation, so species richness tends to decrease in fragments of a given size that are located at a higher elevation, compared to those of the same size but at a lower elevation. When looking at the family-level and in relation to abundance, this trend may differ. For example, at lower elevations the abundance of frogs of the families Craugastoridae and Hylidae tends to increase; these groups of frogs are predominantly tropical in origin [32], while the Plethodontid salamanders are predominantly temperate in origin [32,33] so their abundance tends to be higher at higher elevations. All this suggests that the biogeographic history of the study area has a determining influence on the diversity patterns observed.

The leaf litter can provide adequate levels of moisture and food resources for amphibians to carry out foraging, courtship and egg-laying and other vital activities [34], and in the Veracruz cloud forest it seems particularly important for Craugastorid frogs and Plethodontid salamanders. A similar response by amphibians to litter depth and to the other fragment traits evaluated here has been reported for both nearby Neotropical regions [35–37] and other tropical latitudes [38].

Beta diversity is an important criterion for defining conservation strategies, and in the study region it is a fundamental indicator for conserving amphibian diversity. Our results suggest that compositional dissimilarity and species replacement are a function of landscape heterogeneity, and specifically of differences in fragment area, elevation, litter depth and the geographic distance between fragments. Studies in the same region have detected the total replacement of salamander species between 1000 and 1800 m a.s.l. [39], and high levels of dissimilarity in the composition of frogs, linked to differences in elevation and canopy cover [28]. So, if the goal is to preserve amphibians at the regional level, the forest fragments to be protected must be distributed over a wide range of elevations, and located throughout the entire region (not clustered together). The fragments may be different sizes but should always include some large ones, and can even have low levels of disturbance (because our study fragments did have some signs of disturbance such as solid waste, logging, burning or soil removal). Another important feature in fragmented tropical landscapes, which we did not evaluate here, is the surrounding matrix. This feature has a notable influence on the dynamics and composition of vertebrate communities within forest remnants [40]. In the study region, the forest fragments may be connected biologically by agroecosystems, such as shade coffee plantations [28,30] as well as riparian corridors [41,42], which may act as refuges or facilitate the mobility of amphibians and other biological groups through fragmented landscapes.

The distribution of all the threatened species recorded in this study was not always restricted to only a few sites and their abundance was not always low, in fact they could be considered heterogeneous. Contrary to our expectation, three species were widely distributed and in some cases their abundance was moderate to relatively high (*Craugastor rhodopis*, *Charadrahyla taeniopus* and *Parvimolge townsendi*). The latter is considered to be in imminent danger of extinction by the IUCN [3] and the Alliance for Zero Extinction [1,6], and is a one-of-a-kind species on the verge of extinction according to the EDGE program (edgeofexistence.org). For these species and for the other threatened species—some of which are exclusively distributed in the study area—TMCF fragments are essential to their survival in a mountainous region that has been extensively and drastically transformed. It is worth noting that the salamander *Chiropterotriton* sp. was only recorded in a single fragment, and only one individual was spotted. This suggests, given the sampling effort of almost one thousand person-hours throughout the study, that this species is likely threatened (consider also that 85% of the known species of this genus are threatened). However, more data are needed based on field surveys (i.e. surveying canopy microhabitats, extending sampling effort to the dry season) to be able to provide a reliable assessment of their level of risk (see Sandoval-Comte et al. [29]).

The most biologically rich areas on the planet are unprotected [43], and this region is no exception. There is no federal agency in charge of protecting the cloud forest remnants that still persist in the area [4]. One TMCF fragment is legally protected at the municipal level, the rest are protected by either private or community interests. By increasing the area under this type of voluntary conservation, providing legal support, obtaining funding from NGOs and similar organizations to support the landowners in their efforts, it may be possible to conserve biodiversity in interconnected forest fragments in this region along the lines of Halffter’s [44] "archipelago reserve". These reserves are intended to be regional umbrellas covering as much regional biodiversity as possible through their complementarity and increasing the connectivity between different areas [45]. A priority for The Amphibian Conservation Action Plan developed by the Species Survival Commission of the IUCN, is the addition of conservation area networks that include the distribution ranges of threatened species not currently protected by the existing Protected Areas [46]. The study region could form part of these conservation area networks.

The protection of priority areas such as mountainous central Veracruz would certainly help Mexico’s international commitment to reduce biodiversity loss, renewed in the 2010 Convention on Biological Diversity (www.cbd.int/sp/targets/) in Nagoya.

## Supporting Information

S1 Fig Species accumulation curves and Chao 1 species estimators for all of the data from the ten study sites. Dotted lines are the lower and the upper confidence intervals (at 84%). The number of *singletons* and *doubletons* is shown in the bottom of the graph.
(PDF)

S1 Table Observed and estimated species richness in ten study sites and for all sites together. Completeness is the percent of estimated richness (minimum-maximum).
(PDF)

S2 Table Compositional dissimilarity values between pairs of study sites. a) Total dissimilarity values (β_cc_), b) replacement values (β_-3_) and c) difference in species richness values (β_rich_).
(PDF)

S3 Table Values obtained from the Mantel test. Asterisk indicates significant relationship between environmental variables and the overall compositional dissimilarity, replacement or differences in species richness.
(PDF)
