# Supplementary material for: Amphibian Diversity and Threatened Species in a Severely Transformed Neotropical Region in Mexico
Source: PLoS One. 2015 Mar 23;10(3):e0121652. doi: 10.1371/journal.pone.0121652 (PMC4370706; doi:10.1371/journal.pone.0121652)
Supplement: S1 Fig — Dotted lines are the lower and the upper confidence intervals (at 84%). The number of singletons and doubletons is shown in the bottom of the graph. (PDF) [file pone.0121652.s001.pdf]

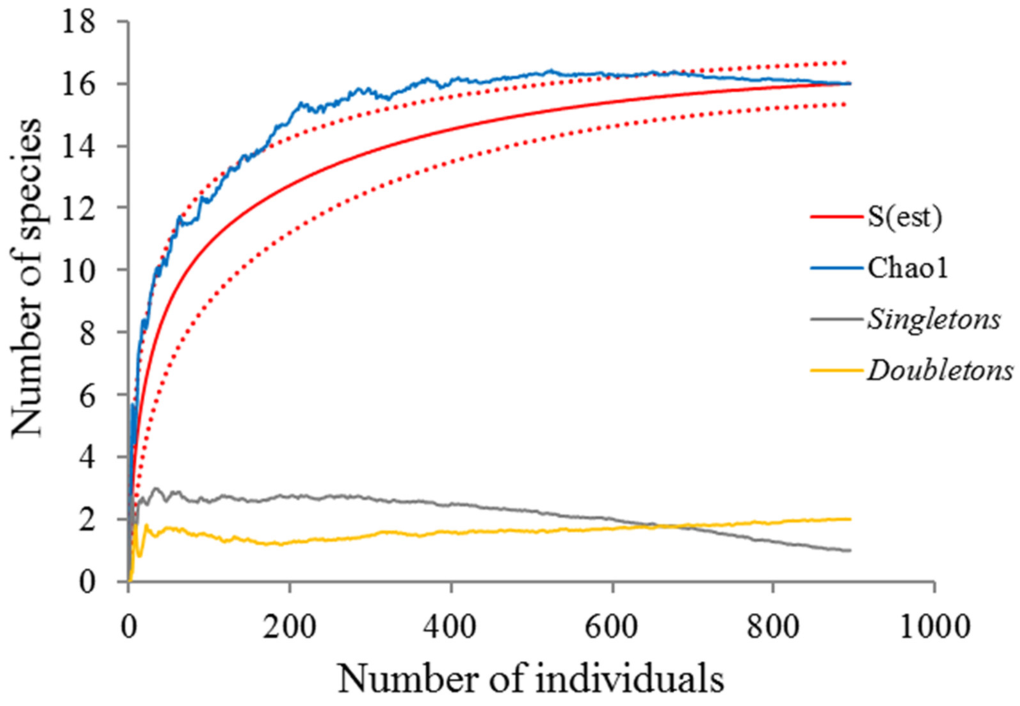

S1 Figure Species accumulation curves and Chao 1 species estimators for all of the data from the ten study sites. Dotted lines are the lower and the upper confidence intervals (at 84%). The number of *singletons* and *doubletons* is shown in the bottom of the graph.
