# Supplementary material for: Amphibian Diversity and Threatened Species in a Severely Transformed Neotropical Region in Mexico
Source: PLoS One. 2015 Mar 23;10(3):e0121652. doi: 10.1371/journal.pone.0121652 (PMC4370706; doi:10.1371/journal.pone.0121652)
Supplement: S1 Table — Completeness is the percent of estimated richness (minimum-maximum). (PDF) [file pone.0121652.s002.pdf]

S1 Table Observed and estimated species richness in ten study sites and for all sites together. Completeness is the percent of estimated richness (minimum-maximum).

Site	Number of species observed	Number of species estimated		Completeness (%)
		S(est)	Chao1	
1	7	8	7	88-100
2	9	10	10	90
3	11	13	14	79-85
4	8	10	10	80
5	6	7	7	86
6	6	6	6	100
7	7	7	7	100
8	6	6	6	100
9	8	8	8	100
10	4	5	5	80
All sites	16	16	16	100
