# Supplementary material for: Amphibian Diversity and Threatened Species in a Severely Transformed Neotropical Region in Mexico
Source: PLoS One. 2015 Mar 23;10(3):e0121652. doi: 10.1371/journal.pone.0121652 (PMC4370706; doi:10.1371/journal.pone.0121652)
Supplement: S2 Table — a) Total dissimilarity values (βcc), b) replacement values (β-3) and c) difference in species richness values (βrich). (PDF) [file pone.0121652.s003.pdf]

S2 Table Compositional dissimilarity values between pairs of study sites. a) Total dissimilarity values (β_{cc}), b) replacement values (β_{-3}) and c) difference in species richness values (β_{rich}).

a)

	Site 1	Site 2	Site 3	Site 4	Site 5	Site 6	Site 7	Site 8	Site 9	Site 10
Site 1		0.22	0.50	0.64	0.56	0.56	0.44	0.56	0.64	0.43
Site 2			0.33	0.58	0.50	0.50	0.40	0.64	0.58	0.56
Site 3				0.27	0.45	0.45	0.36	0.67	0.64	0.64
Site 4					0.44	0.44	0.50	0.73	0.67	0.67
Site 5						0	0.14	0.67	0.73	0.57
Site 6							0.14	0.67	0.73	0.57
Site 7								0.70	0.75	0.63
Site 8									0.60	0.57
Site 9										0.67
Site 10										

b)

	Site 1	Site 2	Site 3	Site 4	Site 5	Site 6	Site 7	Site 8	Site 9	Site 10
Site 1		0.00	0.17	0.55	0.44	0.44	0.44	0.44	0.55	0.00
Site 2			0.17	0.50	0.20	0.20	0.20	0.36	0.50	0.00
Site 3				0.00	0.00	0.00	0.00	0.33	0.43	0.00
Site 4					0.22	0.22	0.40	0.55	0.67	0.22
Site 5						0	0.00	0.67	0.55	0.29
Site 6							0.00	0.67	0.55	0.29
Site 7								0.60	0.67	0.25
Site 8									0.40	0.29
Site 9										0.22
Site 10										

c)

	Site 1	Site 2	Site 3	Site 4	Site 5	Site 6	Site 7	Site 8	Site 9	Site 10
Site 1		0.22	0.33	0.09	0.11	0.11	0.00	0.11	0.09	0.43
Site 2			0.17	0.08	0.30	0.30	0.20	0.27	0.08	0.56
Site 3				0.27	0.45	0.45	0.36	0.33	0.21	0.64
Site 4					0.22	0.22	0.10	0.18	0.00	0.44
Site 5						0	0.14	0.00	0.18	0.29
Site 6							0.14	0.00	0.18	0.29
Site 7								0.10	0.08	0.38
Site 8									0.20	0.29
Site 9										0.44
Site 10										
