# Supplementary material for: Amphibian Diversity and Threatened Species in a Severely Transformed Neotropical Region in Mexico
Source: PLoS One. 2015 Mar 23;10(3):e0121652. doi: 10.1371/journal.pone.0121652 (PMC4370706; doi:10.1371/journal.pone.0121652)
Supplement: S3 Table — Asterisk indicates significant relationship between environmental variables and the overall compositional dissimilarity, replacement or differences in species richness. (PDF) [file pone.0121652.s004.pdf]

**S3 Table** Values obtained from the Mantel test. Asterisk indicates significant relationship between environmental variables and the overall compositional dissimilarity, replacement or differences in species richness.

| | Overall dissimilarity ($\beta_{cc}$) | | | Replacement ($\beta_{-3}$) | | | Differences in species richness ($\beta_{rich}$) | | |
|---|---|---|---|---|---|---|---|---|---|
| Variable | Z-Mantel | r | p | Z-Mantel | r | p | Z-Mantel | r | p |
| Canopy cover | 108.08 | 0.38 | 0.078 | 60.51 | 0.11 | 0.639 | 47.58 | 0.25 | 0.33 |
| Tree height | 37.1 | 0.1 | 0.55 | 21.22 | 0.03 | 0.857 | 15.89 | 0.06 | 0.714 |
| Tree density | 844.3 | -0.14 | 0.489 | 413.41 | -0.33 | 0.132 | 430.89 | 0.32 | 0.157 |
| Basal area | 92.71 | -0.04 | 0.858 | 43.63 | -0.36 | 0.104 | 49.08 | 0.49 | 0.039* |
| Leaf litter | 30.23 | 0.43 | 0.025* | 19.93 | 0.51 | 0.016* | 10.3 | -0.27 | 0.233 |
| Size | 28.81 | 0.53 | 0.015* | 20.06 | 0.58 | 0.018* | 8.75 | -0.26 | 0.316 |
| Shape | 27.5 | -0.03 | 0.857 | 14.51 | -0.18 | 0.351 | 12.99 | 0.22 | 0.262 |
| Elevation | 8444.32 | 0.65 | 0.001* | 5984.89 | 0.72 | 0.001* | 2459.42 | -0.33 | 0.146 |
| Geographical distance | 591.89 | 0.37 | 0.013* | 350.07 | 0.22 | 0.128 | 241.82 | 0.08 | 0.589 |
